# Genomic analysis establishes correlation between growth and laryngeal neuropathy in Thoroughbreds

**DOI:** 10.1186/1471-2164-15-259

**Published:** 2014-04-03

**Authors:** Adam R Boyko, Samantha A Brooks, Ashley Behan-Braman, Marta Castelhano, Elizabeth Corey, Kyle C Oliveira, June E Swinburne, Rory J Todhunter, Zhiwu Zhang, Dorothy M Ainsworth, Norman Edward Robinson

**Affiliations:** 1 Department of Biomedical Sciences, Cornell University, Ithaca, NY 14853, USA; 2 Department of Animal Science, Cornell University, Ithaca, NY 14853, USA; 3 Department of Large Animal Clinical Sciences, Michigan State University, East Lansing, MI 48824, USA; 4 Department of Clinical Sciences, Cornell University, Ithaca, NY 14853, USA; 5 Animal Health Trust, Kentford, Newmarket, UK; 6 Current address: Animal DNA Diagnostics Ltd, William James House Cowley Road, Cambridge CB4 0WX, UK

**Keywords:** Recurrent laryngeal neuropathy (RLN), Thoroughbred, Horse, *Equus caballus*, Genome-wide association study (GWAS), Haplotype, Body size

## Abstract

**Background:**

Equine recurrent laryngeal neuropathy (RLN) is a bilateral mononeuropathy with an unknown pathogenesis that significantly affects performance in Thoroughbreds. A genetic contribution to the pathogenesis of RLN is suggested by the higher prevalence of the condition in offspring of RLN-affected than unaffected stallions. To better understand RLN pathogenesis and its genetic basis, we performed a genome-wide association (GWAS) of 282 RLN-affected and 268 control Thoroughbreds.

**Results:**

We found a significant association of RLN with the *LCORL*/*NCAPG* locus on ECA3 previously shown to affect body size in horses. Using height at the withers of 505 of these horses, we confirmed the strong association of this locus with body size, and demonstrated a significant phenotypic and genetic correlation between height and RLN grade in this cohort. Secondary genetic associations for RLN on ECA18 and X did not correlate with withers height in our cohort, but did contain candidate genes likely influencing muscle physiology and growth: myostatin (*MSTN*) and integral membrane protein 2A (*ITM2A*).

**Conclusions:**

This linkage between body size and RLN suggests that selective breeding to reduce RLN prevalence would likely reduce adult size in this population. However, our results do not preclude the possibility of modifier loci that attenuate RLN risk without reducing size or performance, or that the RLN risk allele is distinct but tightly linked to the body size locus on ECA3. This study is both the largest body size GWAS and the largest RLN GWAS within Thoroughbred horses to date, and suggests that improved understanding of the relationship between genetics, equine growth rate, and RLN prevalence may significantly advance our understanding and management of this disease.

## Background

Equine recurrent laryngeal neuropathy (also known as idiopathic laryngeal hemiplegia) is a common upper respiratory tract disorder of horses and a significant cause of their reduced athletic performance [1]. RLN represents a distal axonopathy of the recurrent laryngeal nerves, which is clinically expressed only on the left side of the larynx. Interestingly, the left nerve is the longest nerve in horses [2,3]. RLN is also a mononeuropathy, as other peripheral nerves of the horse remain unaffected [4]. As a result of the axonal degeneration, secondary paresis to complete paralysis of the intrinsic laryngeal abductor and adductor muscles ensues. Although RLN results in fiber-type grouping in all of the intrinsic laryngeal muscles, it is neurogenic atrophy of one particular abductor, the *Musculus cricoarytenoideus dorsalis* (CAD), that prevents maximal abduction of the arytenoid cartilage and contributes to the development of airway obstruction. The degree of CAD paresis/paralysis is graded endoscopically from 1 to 4 [5] and a significant correlation between the laryngeal grade and the histopathological changes of the CAD has been documented [6].

Recurrent laryngeal neuropathy is an important axonopathy for horses in competitive events, because the resultant paresis of the intrinsic laryngeal muscles leads to obstruction of air flow during intense exercise, an abnormal inspiratory noise known as “roaring,” and, most importantly, reduced performance. Despite its importance, however, the etiology of the axonopathy is unknown. RLN has particular economic importance in Thoroughbred racehorses because these animals enter race training as 2-year-olds. Even though all horses going through sales are endoscopically examined for evidence of RLN, the condition may not yet be clinically evident in some animals [7] so a diagnostic test to predict RLN risk would be very useful.

The prevalence of RLN varies, ranging from between 2-11% in Thoroughbreds [8,9] and 35-46% in the Draft horse breeds [10,11]. In draft breeds, taller horses are at greater risk of developing RLN, but the relationship between height and RLN in Thoroughbreds has not been clearly defined [11,12]. From a genetic point of view, all modern Thoroughbreds originated from three stallions imported into England in the 17^th^ and 18^th^ century that were crossbred to local mares [13]. As a consequence of this restricted founder stock and strong selection for racing performance, modern Thoroughbreds exhibit low genetic diversity and high linkage disequilibrium [14], making them particularly well suited to identify genetic associations using a genome-wide association study (GWAS) approach.

The presence of genetic factors contributing to the pathogenesis of RLN is suggested by the observation that offspring of RLN-affected stallions are more likely to be affected with the disorder than are offspring from unaffected stallions [15,16]. Elucidating the genetic mechanism causing the disease is of particular research interest since, unlike most myelinopathies, RLN manifests itself as a bilateral mononeuropathy [4,17]. However, the genetics of RLN have not been extensively investigated. A GWAS of RLN has been conducted [17] in a mixed population of horses consisting of 234 cases (196 Warmbloods, 20 Trotters, 14 Thoroughbreds, 4 Draft horses) and 228 breed-matched controls. Using a haplotype-based approach based on Illumina Equine SNP50 genotypes, the investigators identified two protective loci in the Warmblood horses on chromosomes 21 (*P* = 1.62 × 10^-6^) and 31 (*P* = 1.69 × 10^-5^) associated with RLN. Neither of these chromosome regions contained regions associated with neuropathies in other mammals or candidate genes that could be immediately linked to peripheral neuropathy. The present study reports on use of a newer Illumina Equine SNP70 BeadChip to conduct a GWAS on a large cohort of Thoroughbred horses that were phenotypically characterized for RLN and height. Specifically, we sought to identify risk loci associated with RLN and in doing so, gain additional insight into the etiopathogenesis of this disease. Because of the clinically reported connection between height and RLN, we also examined their genetic association.

## Methods

### Horses

The protocol for phenotypic characterization and obtaining blood samples and the informed consent forms were approved by the Animal Use and Care Committee of Michigan State University (MSU) and Cornell University. Information on the risks and benefits of the investigation was provided to the horse trainers/owners and informed consent was signed before at the time the sample was taken. Phenotypic data and venous blood samples (collected in 10 ml EDTA tubes) were obtained from 573 Thoroughbred horses evaluated by large-animal specialists from two veterinary teaching hospitals (Michigan State University and Cornell University) and by participating board-certified veterinary surgeons at private practices throughout the US (n = 10). A total of 505 horses at least 2 years old had recorded heights. In each unsedated horse, a videoendoscopic examination of the larynx was performed and a laryngeal grade of 1 to 4 [5] was assigned. This grade was derived following observation of laryngeal movements during eupneic breathing, after induced-swallowing, and after nasal occlusion maneuvers. Cases (RLN-affected), which were largely obtained from participating veterinary surgeons, were horses requiring corrective surgery for RLN. They had laryngeal grades of 3 and 4 (n = 121 and n = 161, respectively) and their median age was 3 years (range 1 to 21 years). Controls, with a median age of 6 years (range 3-24) had laryngeal scores of 1 (n = 268), were obtained from broodmare farms, private stables, veterinary hospital admissions, and two racetracks. Sixteen putative controls received intermediate laryngeal grades (1.5-2.5) and were excluded from the RLN association analysis. With the exception of 3 three year-old horses, all of the control horses were at least 4 years old. All of the control horses were examined by either veterinary teaching hospital faculty or participating veterinary surgeons. Because, in 15% of horses, RLN may grade change later in life [7], a few of the control animals may have developed RLN at a later age, although excluding the 3 three-year old controls did not materially affect the results. Height at withers measurements was reported to the nearest quarter hand (inch) and converted to centimeters.

Samples submitted by practitioners were shipped on dry ice to the Pulmonary Laboratory, MSU and then stored at -20°C. All samples were then processed by personnel in the Cornell Veterinary Biobank [18]. DNA was extracted by use of a DNA purification kit (Gentra Puregene or Qiagen kit), and the concentration (spectrophotometric absorbance at 260 nm) and quality (absorbance ratio: 260 nm/280 nm > 1.80) of each sample was determined. Two μg aliquots of DNA were allocated into 96-well plates and stored at -20°C until samples were genotyped at the Cornell University Biotechnology Resource Center.

### Genotyping

The 573 Thoroughbred samples were genotyped using Illumina Equine SNP70 arrays containing 65,157 markers. Genotype calls were made in GenomeStudio using a GenCall threshold of 0.25. Twenty samples with high missingness were re-run and an additional 29 samples passing QC were run multiple times as technical replicates. A total of 1,470 SNPs were excluded for high missingness (>10%) and 7,751 monomorphic or rare SNPs (minor allele frequency < 0.5%) were excluded. Duplicate concordance at the remaining sites was >99.99%.

For the final dataset, only one array per sample was retained, and an additional seven samples were excluded because their imputed genotypic sex did not match the phenotypic records, indicating a possible misidentification of these samples. Two SNPs (BIEC2_403636 and BIEC2_424368) were excluded because they showed evidence of probe hybridization to multiple genomic locations, and two other SNPs (BIEC2_346860 and BIEC2_840760) were excluded due to low duplicate concordance rates. Additionally, 197 markers were excluded for high heterozygosity (*P* < 1 × 10^-4^ based on Hardy-Weinberg equilibrium) and 24 were excluded for low heterozygosity (*P* < 1 × 10^-32^); filtering was more relaxed for low heterozygosity because population structure would be expected to depress heterozygosity somewhat from Hardy-Weinberg expectation. The final unphased dataset contained 566 horses genotyped at 56,679 markers with a mean call rate of 99.77%. Principal component analysis was computed using Eigenstrat [19] and revealed no evidence of batch effects or significant population stratification in the dataset (Additional file 1: Figure S1). Phenotypic data for these 566 horses are summarized in (see Additional file 2: Table S1).

Genome-wide association was performed using GEMMA [20] after imputation of missing genotypes using ShapeIT v2 [21]. In all cases, association analysis was performed by means of a Wald test using the centered relatedness matrix calculated by GEMMA. However, comparison of these association results with standard association tests (no correction for population structure) revealed very little evidence of *P*-value inflation due to population structure in our cohort (Additional file 3: Figure S2). We used a Bonferroni significance cutoff of 1.16 × 10^-6^, conservatively estimating 43,055 independent comparisons (56,679 total markers less 13,624 markers that are in complete LD, r^2^ > 0.99, in our study) and a family-wise error rate of **α =** 0.05.

To assess association to haplotypes in the ECA3 candidate region a set of SNPs centered on BIEC2_808543 and encompassing approximately 2 Mb (ECA3:104737609–106517004) was selected. We further filtered this set to include only markers with a genotyping rate > 99% and a MAF > 0.01, leaving 45 remaining markers. Linkage disequilibrium between markers was examined using Haploview v4.2 [22]. Haplotype associations to height and RLN status were tested by a general linear model or chi-square test using a sliding window of between 2 and 12 SNPs in PLINK [23]. Regression modeling of the effect of height and putative RLN loci was done with general linear models using R with the package stats (v. 2.15.2) [24].

## Results

### Association mapping of RLN

**Figure 1:**
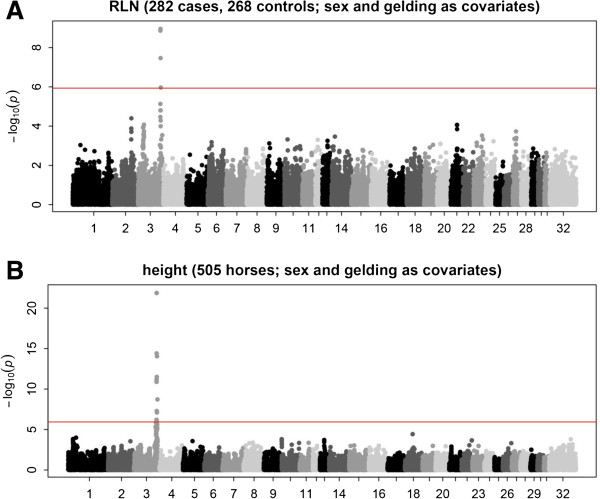
**Manhattan plots of GWAS after accounting for population structure and sex and gelding (see Methods) for RLN and withers height. A**: RLN association in 282 Thoroughbred horse cases and 268 controls. **B**: Height association in 505 Thoroughbred horses.

Using the full case–control dataset (550 horses: 282 cases and 268 controls) and correcting for sex and gelding, the strongest association with RLN was centered on a SNP, BIEC2_808543, adjacent to the ligand-dependent nuclear receptor corepressor-like (*LCORL*) and non-SMC condensing I complex, subunit G (*NCAPG*) genes (*P* = 1.1 × 10^-10^, Figure 1A). Although this locus only explains 6% of the variation in RLN in our cohort, we estimated that our full dataset explained 0.45 ± 0.11 of the variation in RLN score after accounting for sex and gelding. This locus has previously been associated with height in horses [25,26]. Including height as an additional covariate in the model (489 horses: 274 cases and 215 controls) yielded no significant associations after correcting for population structure, although regions of chromosomes 23 (36.2-39.8 Mb) and chromosome 18 (65.8-67.0 Mb showed suggestive associations with RLN (*P* = 5.6 × 10^-6^ and 1.6 × 10^-5^, respectively, Additional file 4: Figure S3A). GWAS for RLN without covariates also yielded an additional suggestive association on chrX at 57.8 Mb (Additional file 4: Figure S3B). The effect of the *LCORL/NCAPG* and chromosome 18 QTLs was consistent between males and females whereas the chromosome 23 QTL appeared stronger in females and the chromosome X QTL appeared stronger in males (Table 1). GWAS for RLN including BIEC2_808543 genotype as a covariate did not reveal any additional associated regions (Additional file 5: Figure S4A).

**Table 1:** Allele frequencies in the cases and controls at the four RLN QTL

		**3:105,547,002**	**18:65,809,482**	**23:39,812,047**	**X:57,820,251**
		**BIEC2_808543**	**BIEC2_417210**	**BIEC2_624338**	**BIEC2_1125869**
		**G/A**	**G/A**	**A/G**	**A/G**
All	Freq aff	0.259	0.323	0.126	0.492
	Freq unaff	0.119	0.457	0.056	0.294
	*P*-value	4.0 × 10^-9^	4.8 × 10^-6^	6.0 × 10^-5^	2.4 × 10^-8^
	Odds ratio	2.58	0.566	2.43	2.32
Males	Freq aff	0.245	0.329	0.107	0.548
	Freq unaff	0.103	0.443	0.057	0.260
	*P*-value	4.3 × 10^-6^	0.0027	0.025	1.8 × 10^-7^
	Odds ratio	2.83	0.616	1.98	3.45
Females	Freq aff	0.299	0.306	0.181	0.410
	Freq unaff	0.135	0.471	0.055	0.310
	*P*-value	5.4 × 10^-5^	0.0011	4.0 × 10^-5^	0.042
	Odds ratio	2.73	0.495	3.81	1.54

### Association mapping of body size

We performed GWAS on height using sex (mare, stallion, and gelding) as a categorical covariate for the 505 horses of at least two years of age with recorded heights (Figure 1B). The strongest association signal (*P* = 1.3 × 10^-22^) centered on the same SNP, BIEC2_808543, in *LCORL/NCAPG* locus that was the strongest association for RLN. All 12 SNPs passing the Bonferroni significance threshold were in this region (ECA3:104,403,770-109,065,924). We estimated that the entire SNP70 panel explains 0.24 ± 0.11 of the variation in withers height after accounting for sex and gelding [20].

Although height differed significantly between mares, geldings, and stallions, sex and gelding only explained 6.9% of the variation in height in the samples. In contrast the *LCORL/NCAPG* minor (G) allele count at BIEC2_808543 had an effect of increasing height 3.7 cm (S.E. = 0.4) and explained 16.6% of the variation in height in the dataset. This effect was roughly additive and consistent in sires, mares, and geldings (Table 2). A model incorporating sex, gelding, and BIEC2_808543 explained 23.7% of the variance in height, and was not improved by adding age to the model (-0.1 ± 0.06 cm/yr, *P* = 0.08). GWAS for height including BIEC2_808543 genotype as a covariate did not reveal any additional associated regions (Additional file 5: Figure S4B).

**Table 2:** **Frequency of *LCORL*/*NCAPG *** **marker BIEC2_808543 on withers height and RLN**

**N**	**AA**	**AG**	**GG**
Stallion	79	41	6
Gelding	157	68	6
Mare	137	64	8
Total	**373**	**173**	**20**
Mean height (cm)			
Stallion	164.7	168.0	170.6
Gelding	162.4	166.7	168.5
Mare	160.7	164.5	169.8
Total	**162.3**	**166.2**	**169.7**
Risk of RLN			
Stallion	0.924	1	1
Gelding	0.313	0.621	0.833
Mare	0.263	0.453	0.875
Total	**0.428**	**0.647**	**0.900**

### Correlated genetic signatures of RLN and height

A binomial regression model showed that height and *LCORL/NCAPG* were strongly correlated with RLN even after accounting for sex and gelding (Table 3). Although the variables were correlated, they were both significant in the full model for RLN, jointly incorporating the effects of sex, gelding, height, and the putative RLN loci on ECA 3, 18, 23 and X (Table 3).

**Table 3:** Effect sizes of height and QTLs on RLN using binomial regression

	***AIC***	***Height***	***LCORL/NCAPG***	***chr18_QTL***	***chr23_QTL***	***chrX_QTL***
Sex + gelding	619.1					
Sex + gelding + height	505.8	1.68				
Sex + gelding + *LCORL/NCAPG*	580.3		1.17			
Sex + gelding + height + *LCORL/NCAPG*	499.1	1.42	0.63			
Sex + gelding + height + 4 loci	444.8	1.61	0.64	-0.85	0.60	1.46
*P*-value (full model)		1.3 × 10^-8^	0.0067	5.2 × 10^-6^	1.3 × 10^-5^	3.6 × 10^-5^

P-values are given for the full model incorporating sex, gelding, height, and genetic effects.

Because of the similar GWAS results for height and RLN, we used bivariate restricted maximum likelihood (REML) analysis [27] to estimate the genetic correlation between height and quantitative RLN score. Bivariate REML estimates of “chip heritability” after accounting for sex and gelding were remarkably similar to our LMM estimates (0.22 and 0.46 for height and RLN, respectively), and the estimated genomic correlation between height and RLN was nearly complete (0.98 ± 0.19). Chromosomes 3 and X explained the largest proportion of the variance in height and were also significantly associated with RLN severity, although other chromosomes also show elevated associations with RLN (e.g., ECA6 and ECA9, see Additional file 6: Figure S5).

**Figure 2:**
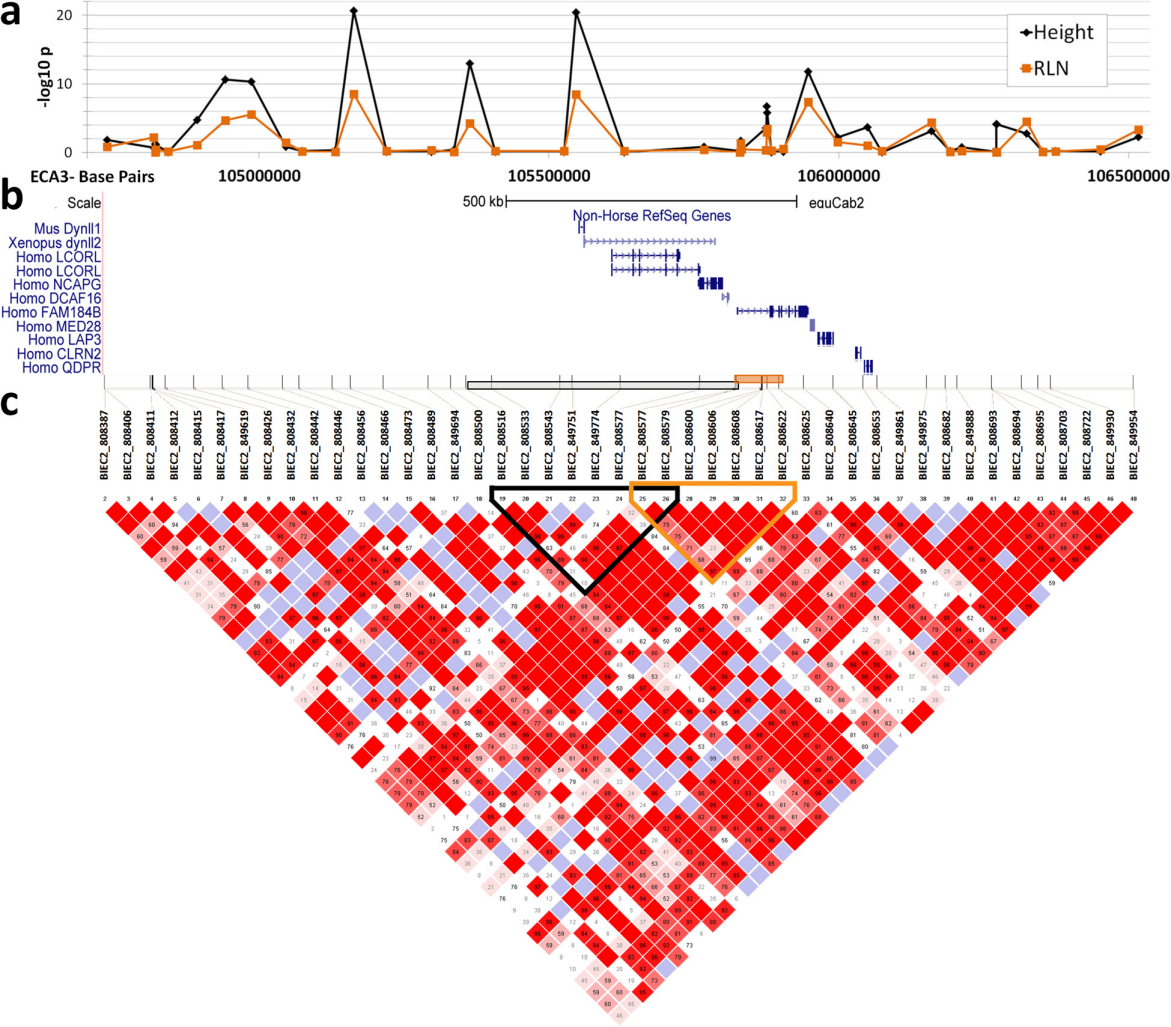
**Single marker association tests (A.) followed by haplotype analysis (C.) reveals different, but overlapping blocks correlated with RLN (orange) and height (black).** Candidate genes *LCORL* and *NCAPG* are encompassed by the height haplotype, while a portion of the *FAM184B* gene lies within the RLN associated haplotype **(B)**. Adapted from the UCSC Genome Browser.

Haplotype analysis revealed that height is associated with an eight SNP block spanning ECA3: 105363241–105830625 (uncorrected *P* = 8.18 × 10^-23^). This genomic region includes both the *LCORL* and *NCPG* genes (Figure 2). The haplotype generating the smallest p-value for RLN is a seven SNP block overlapping with the height block by two markers (uncorrected *P* = 6.29 × 10^-11^) (see Additional file 7: Table S2 and Additional file 8: Table S3). This block spans ECA3: 105829484–105884170 and includes the 5’ half of the *FAM184B* gene (Figure 2). Examination of the 1132 chromosomes phased using ShapeIT revealed that 209 chromosomes bear both the RLN and height-associated haplotype blocks. Only one chromosome possessed the height block but not the RLN haplotype. 81 chromosomes contained the RLN haplotype but not the height associated block, leaving 841 chromosomes possessing only other haplotypes.

## Discussion

The selective breeding of domestic animals for desirable traits has often been accompanied by the appearance of genetically determined undesirable characteristics: examples include malignant hyperthermia in swine selected for rapid increase in muscle mass, osteoporosis in chickens selected for high rates of egg production, and hyperkalemic periodic paralysis in Quarterhorses selected for muscular appearance. In general, horses have been selectively bred for power and speed. The former requires size and muscle mass while the latter requires height (long legs), a compact muscle mass close to the trunk, and high rates of maximal oxygen consumption. Although selection for height apparently has been accompanied by an increased prevalence of RLN, the genetic basis for this is currently unknown.

The current study, which used the largest cohort of RLN-affected and unaffected horses ever assembled from a single breed revealed a very strong genetic correlation between RLN and height. This association was previously well known across breeds (ponies are rarely clinically affected by RLN and draft horses frequently are), but has never been demonstrated over the small range of heights (152 to 178 cm) spanned in the present study. This was evident not only in the clinical observations (cases were taller than controls), but also in the GWAS results. The haplotype associations for height and RLN were coincident and the genetic correlation between the traits was >90%.

The phenotype of RLN was defined based on the presence (cases) or absence (controls) of endoscopically visible failure of abduction of the left arytenoid and associated vocal fold. This clinical and functional definition was used because veterinarians in the field use it and because it has important implications for athletic performance. There were two reasons for selection of grade 3 as the threshold score for a case: first, horses with this grade are unable to maintain vocal fold abduction and therefore require surgical correction to restore their performance; and second, failure of abduction is easily recognized endoscopically by experienced veterinarians (those participating in selection of cases examine many racehorses before sale as well as clinical cases). However, histopathological examination of the intrinsic muscles of the larynx can reveal muscle fiber-type regrouping (7evidence of axon death and reinnervation from a neighboring axon) in some horses that do not have clinical evidence of laryngeal paresis [28] and therefore would be controls under the definition used in the present investigation. Furthermore, foals can demonstrate fiber-type regrouping at birth [29] and ponies have fiber-type regrouping but rarely develop the clinical syndrome. Definition of cases and controls based on the presence or absence of fiber-type regrouping is not currently practical, and it is possible that clinical expression of RLN involves factors other than just changes in muscle fiber type.

Recently, several independent studies have documented strong associations between the *LCORL/NCAPG* region and body size in the horse [30,31], but no conclusive causative mutation has been detected to date. A role for both *LCORL* and *NCAPG* in growth is supported by well-validated associations to human height [32,33], and to fetal growth rate in cattle [34].

Within the Thoroughbred breed, the ECA3 locus appears to have the single largest effect on withers height (Figure 1B). Previous work in 16 diverse breeds selected for extreme body size identified three significant loci in addition to ECA3 (*HMGA2*, *ZFAT,* and *LASP1*) as well as a Thoroughbred-specific QTL on ECA28, but no signal for these loci was detected in this dataset [26]. Although marker coverage for *HMGA2* is poor on the Illumina SNP70 product, the same markers for *ZFAT* are included in this analysis and segregate at ~ 20% in our cohort, but do not appear to impact withers height. The association of *ZFAT* with height is replicated in a study of 1,077 Franches-Montagnes horses [25], suggesting the effect of the size QTL near *ZFAT* may be dependent on genetic background. The previous estimate that 59% of the variation in height in Thoroughbreds was explained by *LCORL*/*NCAPG* and the QTL on ECA28 is significantly higher than our estimate of 24% based on the entire SNP dataset. This may reflect an overestimation in their analysis due to small sample size (N = 48) or an underestimation in ours due to the small range of Thoroughbred heights, environmental variability, and/or measurement error.

Our study confirms anecdotal evidence and previous reports in draft breeds identifying an association between RLN and large size in horses [11,12]. However, the nature of this association remains unknown. Certainly, large body size will increase the overall length of the recurrent laryngeal nerve, perhaps increasing the risk for mechanical injury of the nerve during athletic performance or for damage associated with defects in axonal transport. Alternatively, *LCORL* and *NCAPG* are genes encoding transcription factors and could have diverse targets with varying physiological functions. Thus, the same causative mutation may be implicated for both body size and RLN phenotypes. Lastly, given extensive LD across the ECA3 locus, RLN and body size may be influenced by two independent genetic changes associated by linkage. Further work will be needed to distinguish which of these three hypotheses is the most likely explanation for the association between this ECA3 locus and RLN.

Besides the *LCORL/NCAPG* locus, the chromosome 18 and X candidate loci are near to genes that are likely to affect muscle physiology and growth in horses (*MSTN* and *ITM2A*). Interestingly, the primary association signal in [17] is also located in a myosin gene (*MYO9B*), a candidate gene that may be very relevant to the pathology of a distal axonopathy like RLN. Yet, our study suggests that QTLs on chromosome 21 and 31 identified by Dupuis *et al.*[17] are not an important influence on RLN in our Thoroughbred cohort. Genetic background may play an important role in a polygenic and multifactorial condition like RLN. Environmental conditions are also drastically different in the actively training racehorse (the majority of animals sampled for this work) and Warmblood horses used primarily for the sports of show jumping and dressage. Mean age of animals between these two studies was also quite different. Cases used here were on average just 3 years of age versus ~8 years of age in the Dupuis study ([17]; Dupuis personal communication). Therefore, the ECA3 locus may be most important for development of RLN in young horses in training for near maximal athletic performance.

## Conclusions

The results of this study add further confirmation to the association between height and the clinical occurrence of RLN and indicate the close relationship between the genetic loci determining these two characteristics. However, despite being statistically significant, LCORL/NCAPG accounted for only 6% of the variation in RLN. However, in these Thoroughbreds, the proportion of variation of RLN explained by the entire SNP70 set (“chip heritability”) was 45%, which, although much higher than in most complex diseases [35], suggests that multimarker tests will be needed for accurate prediction of RLN risk. Because of the nearly complete genetic correlation between height and RLN risk in Thoroughbreds, it may be difficult reduce RLN prevalence without altering body size or other growth characteristics that may be important for racing performance. However, our results do not preclude the possibility of modifier loci that attenuate RLN risk without reducing size or performance, or that the RLN risk allele is distinct but tightly linked to the body size locus on ECA3.

## Competing interests

Some of the information in this paper has been used in a patent application.

## Authors’ contributions

ARB and SAB participated heavily in experimental design, data analysis, and manuscript preparation. ALB was instrumental in establishing the cohort of samples. EC prepared DNA samples, established the DNA bank, and participated in data analysis. KCO, RJT and ZZ participated in experimental design and data analysis. MC prepared samples and was involved in data analysis. DMA contributed samples to the cohort, was involved in data analysis and manuscript preparation. JES wrote the first plan to establish the cohort and conducted the genetic analysis (she is now in private industry). NER (the corresponding author) assisted JES with the initial experimental design, oversaw and participated in collecting the majority of the samples for the cohort, participated in discussions of data, and prepared the final manuscript submission. All authors reviewed the final manuscript.

## Supplementary Material

Additional file 1: Figure S1 Principal component analysis (PCA) of genotype data shows no stratification according to RLN status or evidence for significant population substructure within the cohort. Red = RLN affected, black = control. Click here for file

Additional file 2: Table S1 Summary of horses used for height and RLN association mapping. Click here for file

Additional file 3: Figure S2 QQ-plot for RLN (A) and height (B). There is little genomic inflation of P-values in the data in uncorrected PLINK (gray) or population structure corrected GEMMA (black) association scans for height (B) in our dataset. The top dozen SNPs are all linked to the ECA3 locus near *LCORL/NCAPG*. The RLN association (A) is shown with sex as the covariate in GEMMA (Figure 1B). Genomic inflation (γ) is 1.0058. Click here for file

Additional file 4: Figure S3 A: GWAS of RLN using sex, gelding, and height as covariates yields additional suggestive associations on ECA18 and ECA23 (489 horses: 274 cases and 215 controls). B: GWAS of RLN without covariates yields an additional signal on chromosome X. Click here for file

Additional file 5: Figure S4 Genome-wide association of RLN (A) and height (B) using sex, gelding, and BIEC2_808543 (*LCORL/NCAPG*) allele count as covariates shows no other significant genetic associations with these traits. Click here for file

Additional file 6: Figure S5 Partitioning the variance explained by each chromosome for height (x-axis) and RLN grade (y-axis) in our sample (550 horses) after accounting for sex and gelding as covariates. The variance explained for RLN case/control was not estimable by the REML method with sex and gelding covariates; therefore, RLN grade (1-4) was used instead. REML estimated total variance explained genome-wide for height and RLN grade is 59% and 61%, respectively, somewhat higher than the estimates obtained by the LMM method or by bivariate REML (see Methods). Click here for file

Additional file 7: Table S2 P-values resulting from a haplotype association test for withers height. Click here for file

Additional file 8: Table S3 P-values resulting from a haplotype association test for RLN status (case/control). Click here for file
