# Supplementary figures and images for: Genomic analysis establishes correlation between growth and laryngeal neuropathy in Thoroughbreds
Source: BMC Genomics. 2014 Apr 3;15:259. doi: 10.1186/1471-2164-15-259 (PMC4051171; doi:10.1186/1471-2164-15-259)

## Slide 1
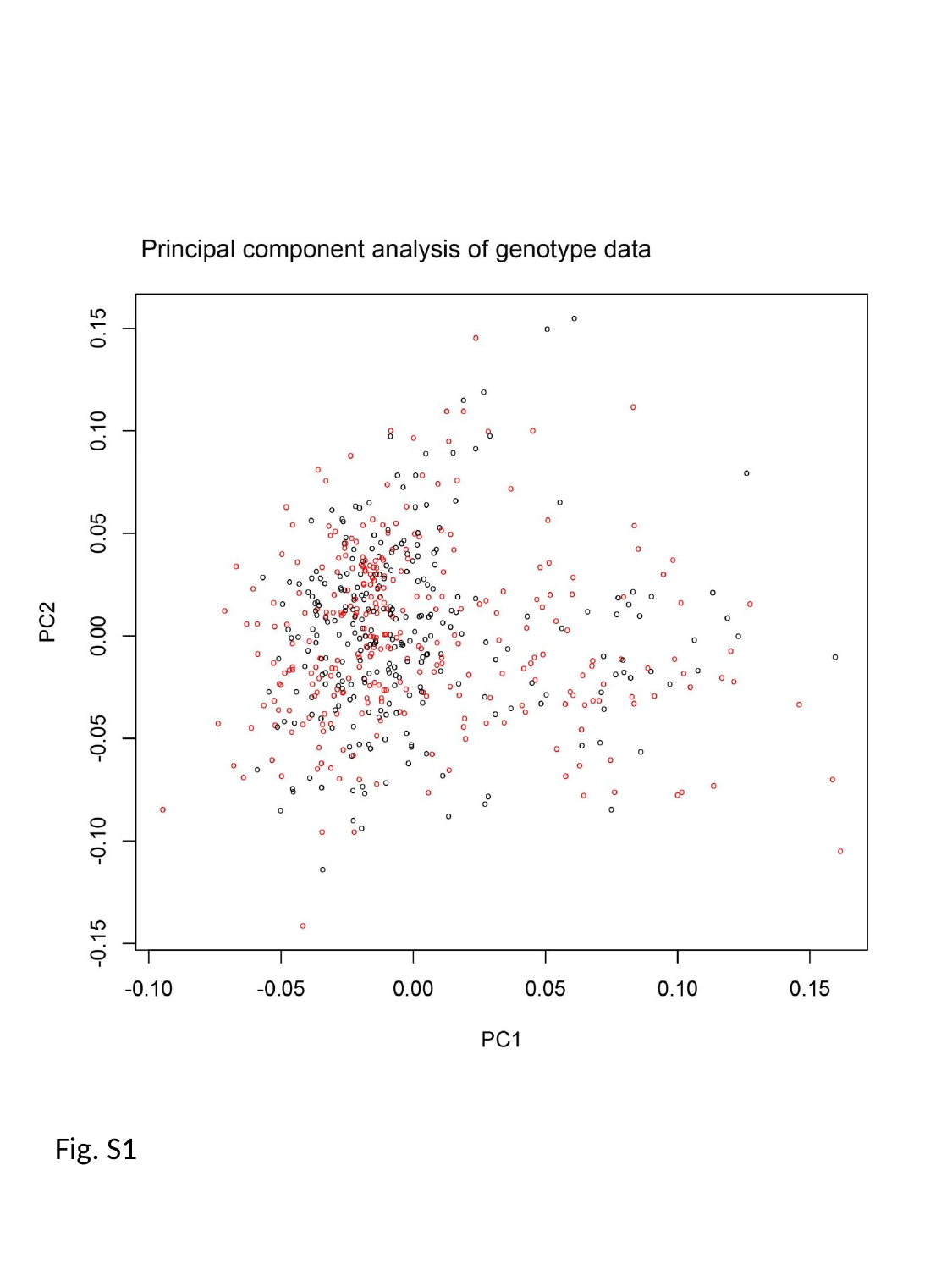

Fig. S1

Supplement: Additional file 1: Figure S1 — Principal component analysis (PCA) of genotype data shows no stratification according to RLN status or evidence for significant population substructure within the cohort. Red = RLN affected, black = control. [file 1471-2164-15-259-S1.PPTX]

## Slide 1
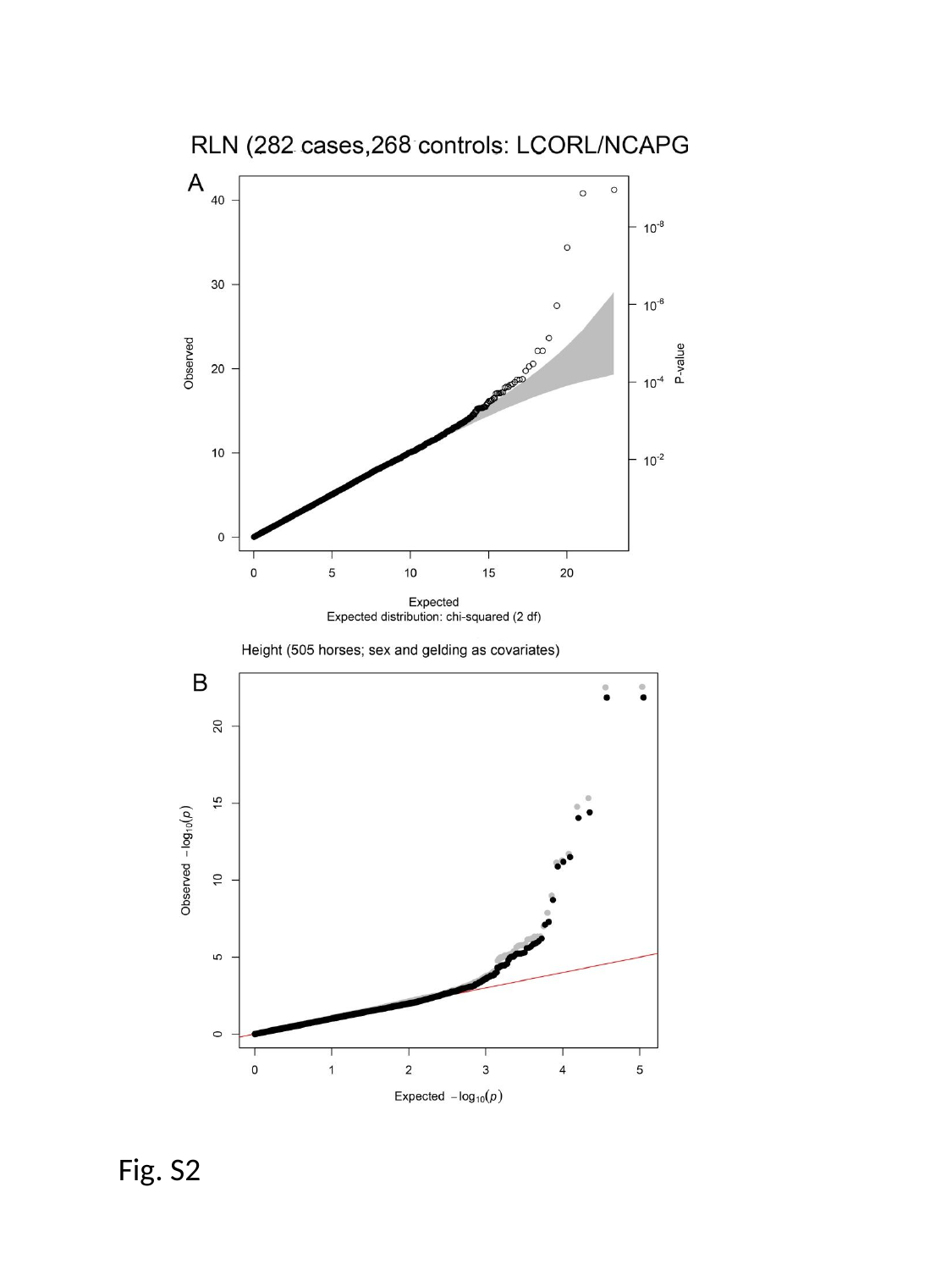

Fig. S2

Supplement: Additional file 3: Figure S2 — QQ-plot for RLN (A) and height (B). There is little genomic inflation of P-values in the data in uncorrected PLINK (gray) or population structure corrected GEMMA (black) association scans for height (B) in our dataset. The top dozen SNPs are all linked to the ECA3 locus near LCORL/NCAPG. The RLN association (A) is shown with sex as the covariate in GEMMA (Figure 1B). Genomic inflation (γ) is 1.0058. [file 1471-2164-15-259-S3.PPTX]

## Slide 1
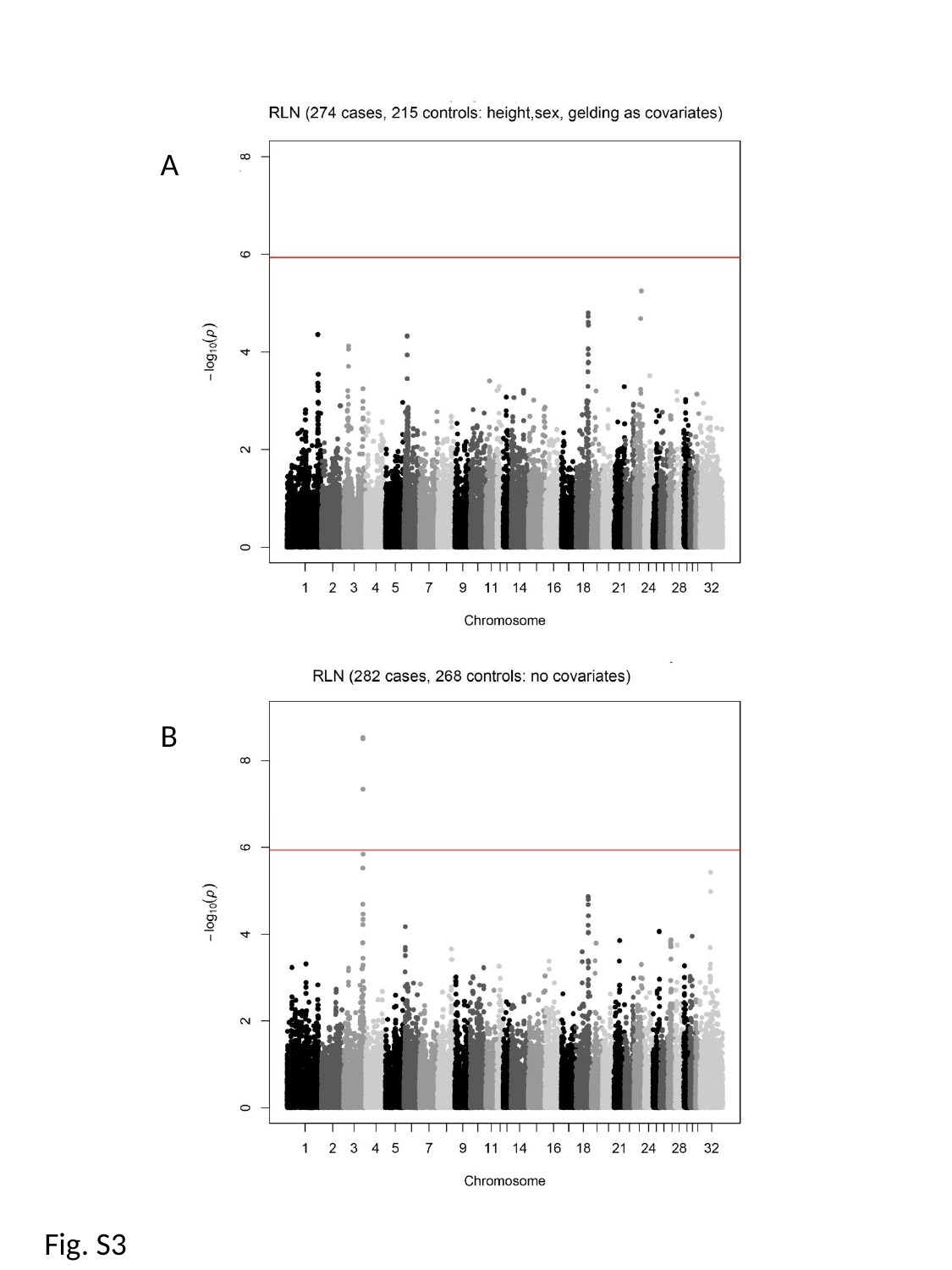

A
B
Fig. S3

Supplement: Additional file 4: Figure S3 — A: GWAS of RLN using sex, gelding, and height as covariates yields additional suggestive associations on ECA18 and ECA23 (489 horses: 274 cases and 215 controls). B: GWAS of RLN without covariates yields an additional signal on chromosome X. [file 1471-2164-15-259-S4.PPTX]

## Slide 1
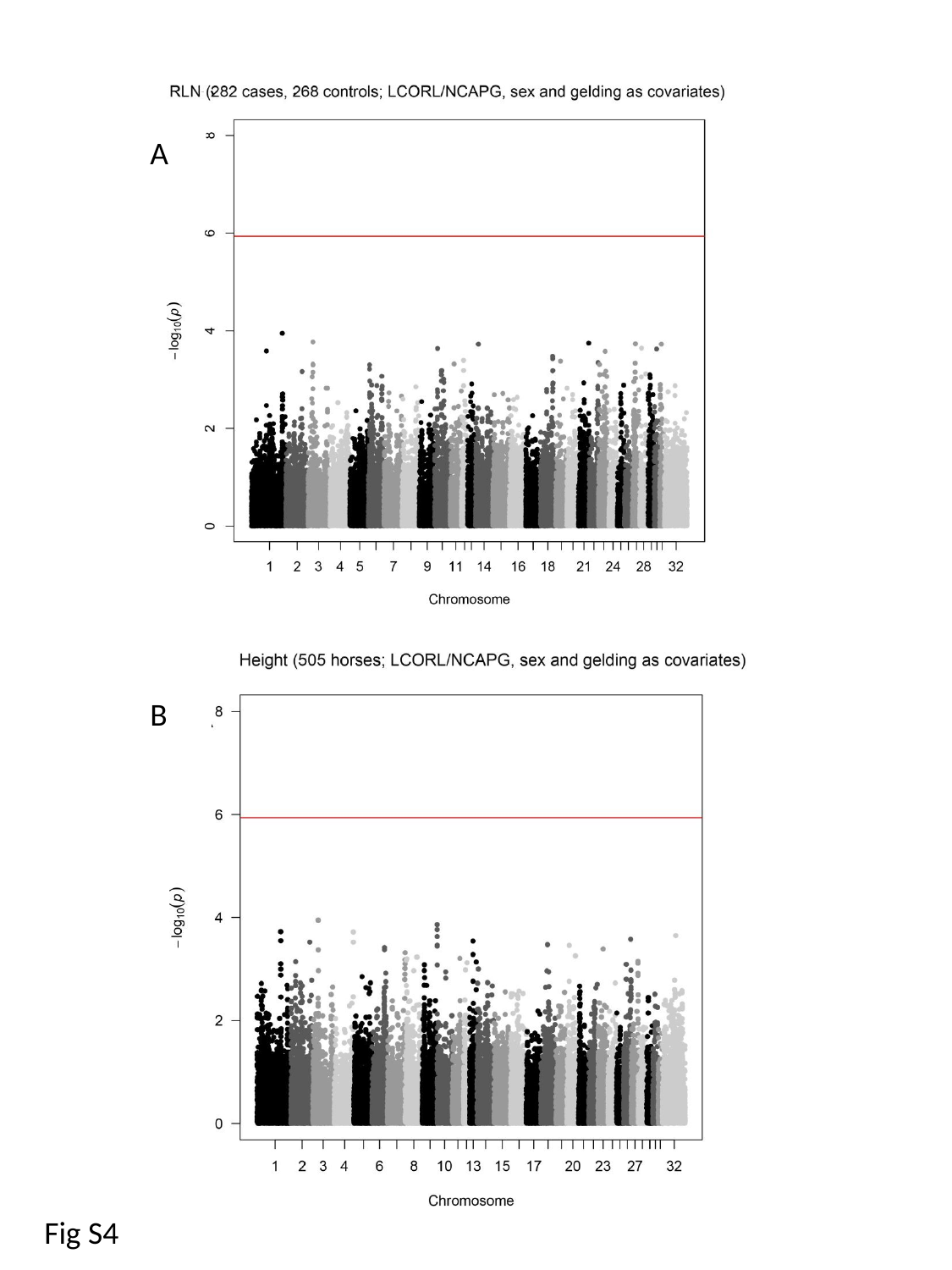

A
B
Fig S4

Supplement: Additional file 5: Figure S4 — Genome-wide association of RLN (A) and height (B) using sex, gelding, and BIEC2_808543 (LCORL/NCAPG) allele count as covariates shows no other significant genetic associations with these traits. [file 1471-2164-15-259-S5.PPTX]

## Slide 1
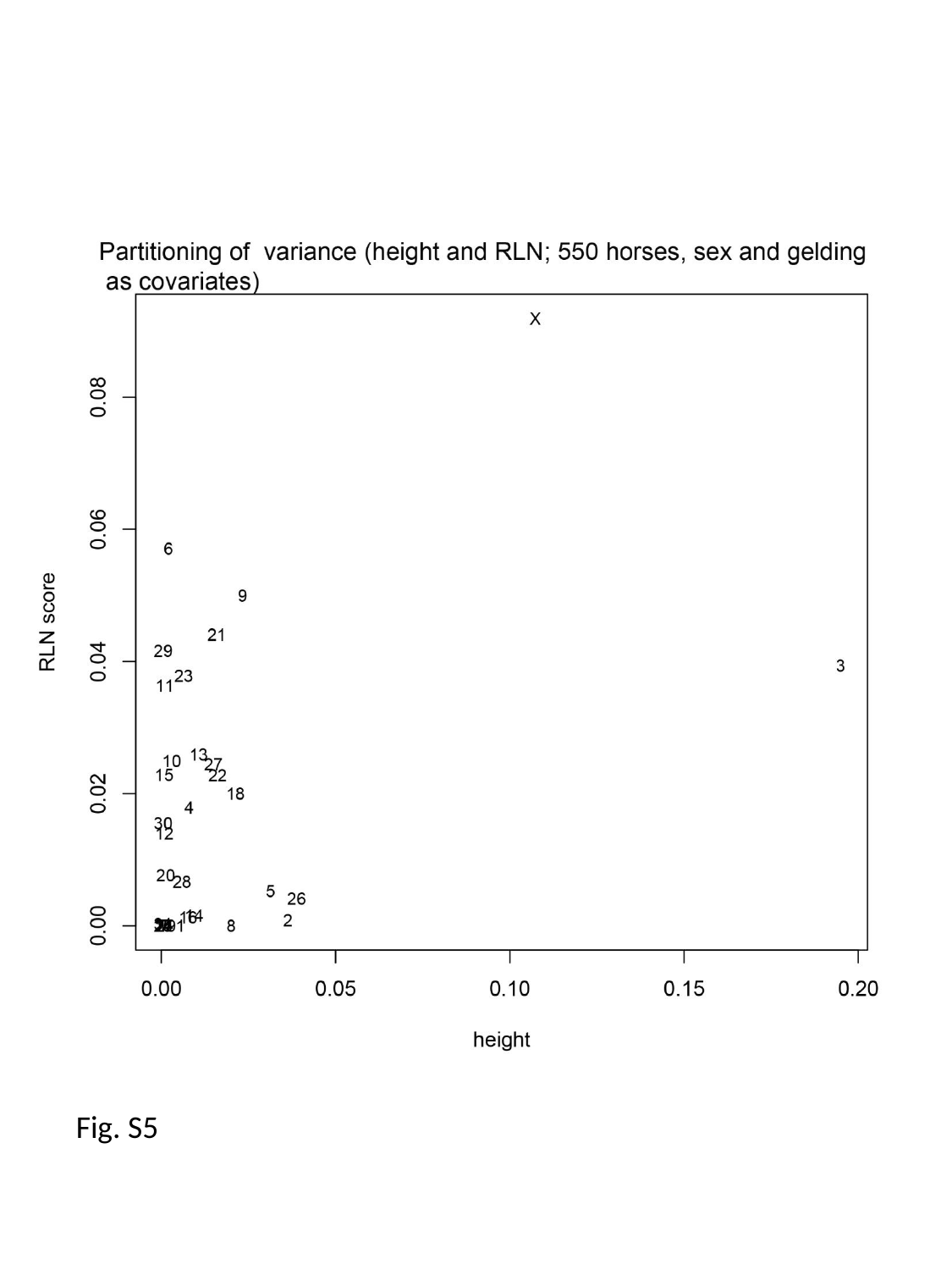

Fig. S5

Supplement: Additional file 6: Figure S5 — Partitioning the variance explained by each chromosome for height (x-axis) and RLN grade (y-axis) in our sample (550 horses) after accounting for sex and gelding as covariates. The variance explained for RLN case/control was not estimable by the REML method with sex and gelding covariates; therefore, RLN grade (1-4) was used instead. REML estimated total variance explained genome-wide for height and RLN grade is 59% and 61%, respectively, somewhat higher than the estimates obtained by the LMM method or by bivariate REML (see Methods). [file 1471-2164-15-259-S6.PPTX]
