# Supplementary material for: Genomic analysis establishes correlation between growth and laryngeal neuropathy in Thoroughbreds
Source: BMC Genomics. 2014 Apr 3;15:259. doi: 10.1186/1471-2164-15-259 (PMC4051171; doi:10.1186/1471-2164-15-259)
Supplement: Additional file 2: Table S1 — Summary of horses used for height and RLN association mapping. [file 1471-2164-15-259-S2.DOCX]

Table S1. Summary of horses used for height and RLN association mapping.

| N cases | | N  controls | N  intermediate | mean ± sd height (cm) | mean (range) age (y) |
| --- | --- | --- | --- | --- | --- |
| sires | 119 | 6 | 1 | 166.1 ± 4.2 | 3.3 (1 - 15) |
| geldings | 91 | 125 | 15 | 163.8 ± 5.3 | 6.3 (2 - 24) |
| mares | 72 | 137 | 0 | 162.4 ± 5.1 | 5.8 (1 - 20) |
